# Effect of Particle Size and Constraint Conditions on Single Particle Strength of Carbonate Sand

**DOI:** 10.3390/s22030765

**Published:** 2022-01-20

**Authors:** Yong He, Guojun Cai, Lei Gao, Huan He

**Affiliations:** 1 School of Transportation, Southeast University, Nanjing 210000, China; hy02150350@126.com (Y.H.); focuscai@163.com (G.C.); 2 College of Civil and Transportation Engineering, Hohai University, Nanjing 210000, China; gaoleihhu@hhu.edu.cn

**Keywords:** particle crushing, carbonate sand, coordination number, particle shape

## Abstract

Carbonate sand is often encountered and utilized as construction material in offshore engineering projects. Carbonate sand particles, which are porous and angular, are found to be highly crushable under high stress conditions, whereas the mechanisms and controlling factors for the crushing of carbonate sand particles are not well developed. The crushability and particle strength of around 400 particles from three fractions (5–10 mm, 2–5 mm, and 1–2 mm) of carbonate sand from the South China Sea were investigated via grain-scale single particle crushing tests. Special emphasis was placed on the effect of external constraint conditions (i.e., coordination number) and intrinsic particle morphology characteristics on the particle strength of carbonate soil. The particle strength of carbonate sand was found to be around half of quartz sand in terms of characteristic stress. Negative correlations, which could be depicted by an exponential equation, were found between the particle size and particle strength. Due to elongated particle shape and tensile stress concentration, a higher coordination number may lower the particle strength, which contradicts what was reported for quartz sands. A series of seven fundamental particle dimensions and five particle shape descriptors was characterized, and the aspect ratio was found to be one of the more influential shape descriptors for particle strength. The results enriched the database for the analysis of highly irregular geomaterial and provided insights into controlling factors of particle strength and crushing mechanisms of the carbonate sand.

## 1. Introduction

In tropical regions, carbonate sand is often encountered and/or utilized as construction material in coastal and offshore construction projects, such as marine oil platforms, offshore wind turbine construction, and artificial islands hydraulic reclamation [1,2,3,4,5,6,7,8,9,10]. The strength and stress–strain behavior of the foundation soil are among the most critical engineering properties that need to be determined for the safe design and construction of infrastructures. However, many studies in the literature emphasized that carbonate sand particles can be easily crushable in comparison with regular quartz sands, and the crushing of particles under high stress can alter mechanical and physical properties notably, such as changes in grading, high magnitude of compressibility and volume change, unique shearing behavior, low permeability, and high thermal conductivity [4,11,12,13,14,15,16,17]. High stress conditions can be common in projects such as driven piles or foundations of gravity hydraulic structures; thus, particle crushing characteristics, especially for crushable sand, are important for investigation.

Researchers noticed the significance of particle breakage for macro-scale behavior back in the middle of the twentieth century [18,19,20], and many researchers investigated the effect of particle breakage on compression and shearing behavior by applying element testing, i.e., on the mesoscale, thereafter [4,11,13,21,22]. The amount of particle breakage was found to be closely related to external factors, including stress magnitude [11], shearing strain [4], stress path [22], saturation condition [23], and the duration of the load [13,24].

Apart from external conditions, researchers in recent decades investigated particle strength from grain scales and found that the intrinsic properties of granular material at the particle scale, such as particle shape, particle size, coordination number, and mineralogy, are the fundamental physical properties that influence the susceptibility of the particle crushing [22,25,26,27,28,29,30,31]. Nakata et al. [25] and Wang and Coop [29] reported the negative correlation between particle strength and particle size by testing quartz sands; Nakata et al. [25] reported that the size effect was not obvious for felspar particles, and the difference between quartz sand and felspar particles is due to their difference in particle composition and microstructures. The magnitude of local roundness at the contact between the particle and the loading platen was found to affect the particle failure mode positively for Leighton Buzzard sand and completely decomposed granite particles, whereas two-dimensional particle shape parameters, including the sphericity and roundness, had limited effect on particle strength [29]. Extending two-dimensional particle shape parameters into three-dimensional analysis, Zhou et al. [31] conducted a series of combined finite-discrete element method (FDEM) numerical experiments and reported that the particle strength increased with an increase in particle sphericity, roundness, and local roundness. Todisco et al. [30] stressed that material hardness can be of key importance for the particle crushing mode and particle strength since it affects local deformation at the contacts. There was a general increase in particle strength when the coordination number increased, but more tests and analyses are required due to the scatter of data, as mentioned by Todisco et al. [28] and Todisco et al. [30]. From the literature, it can be concluded that the influencing factors, as well as the mechanisms, for particle strength are still not well explored; thus, a much larger database is desirable for exploring the variety of particulate geomaterials. Despite being one of the most crushable granular materials encountered in engineering projects, only a few works investigated the particle strength of carbonate soil at grain scale.

In this study, the particle shape characteristics and particle strength of a carbonate sand from the South China Sea were investigated via an experimental approach. The particle strength of carbonate sand particles from three fractions was studied under different particle contact conditions. A range of particle shape characteristics was quantified for the particles, and special emphasis was placed on the investigation of the influence of particle size, morphology, and contact condition on particle strength.

## 2. Materials and Methods

### 2.1. Materials

**Figure 1:**
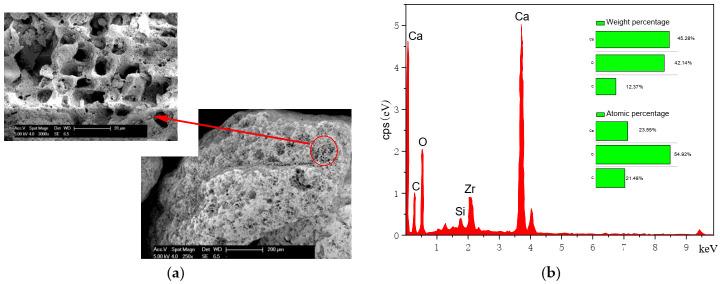
(**a**) Representative scanning electron microscope images of carbonate sand; (**b**) EDS results.

Carbonate sand from the South China Sea was adopted for the investigation of particle shape and particle strength characteristics. The original material was sieved to obtain three uniform fractions for further experiments. Typical scanning electron microscope (SEM) images of a particle from 2 to 5 mm fraction are provided in Figure 1a, where it can be observed that sand particles are irregular in shape with internal pours covering the majority of the surface area. An energy dispersive spectroscopy (EDS) test was also conducted, and the results presented in Figure 1b show that the weight percentage of calcium is 45.28%, which manifests that the dominant compound of carbonate sand is calcium carbonate.

### 2.2. Apparatus and Testing Procedures

**Figure 2:**
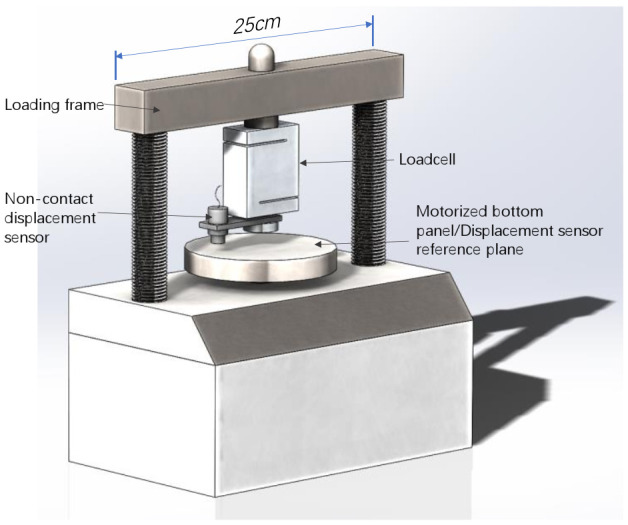
Schematic illustration of the testing apparatus.

The single-particle loading apparatus was custom modified from an unconfined compression testing apparatus, as shown in Figure 2. The loading arm, which is mounted on the high stiffness reacting frame, was redesigned and custom manufactured to equip a high accuracy S-beam type loadcell with a capacity of 500 N and an accuracy of 0.1 N. A non-contact eddy current displacement sensor with a measuring range of 6 mm and an accuracy of 0.001 mm was used. The adoption of the non-contact displacement sensor, compared to traditional linearly variable displacement transformers, avoids possible influence on force measurement while maximizing the accuracy of the load–displacement relationship captured.

Experimental procedures can be divided into two steps: acquisition of particle images and multiple contact compression tests, which are then followed by an analysis that extracts basic size parameters and failure stress of particles.

(1) Acquisition of particle images. Carbonate sand particles were washed with clean water and dried for 48 h at a temperature of 70 °C to prevent fine particles from adhering to the surface or inner pores of coarse particles, which could result in measurement errors. Then, particles are photographed simultaneously from two mutually perpendicular directions using a digital microscope (supplied by Dino-Lite).

(2) Multiple contact compression tests. During the test, the motorized bottom panel would be pushed upwards at a velocity of 0.1 mm/min to apply compressive force onto the specimen, and two digital microscopes were set in orthogonal directions to observe specimens and record the testing process. The force and displacement measured are collected in real time by using a data acquisition unit (supplied by the National instrument), and the results are monitored and recorded by a custom-written program in LabVIEW. Two digital microscopes continuously record the particle compression process at 50 frames per second from two perpendicular directions for observation. The test was terminated when particle structural failure was reached, which was judged by the force–displacement relationship recorded and visual observation.

### 2.3. Testing Program

**Figure 3:**
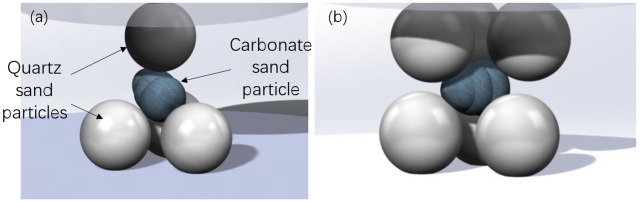
Schematic illustration of high coordination number test setups: (**a**) coordination number equal to 4 (CN4); (**b**) coordination number equal to 6 (CN6).

**Figure 4:**
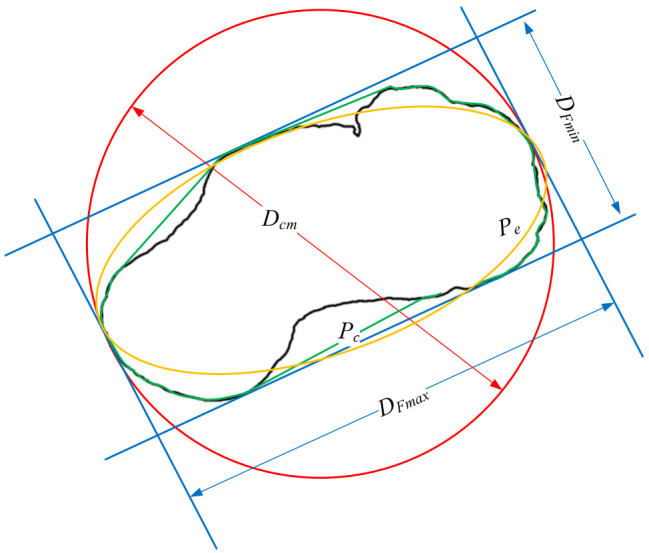
Particle fundamental dimensions analysis of a representative specimen.

Characterization and quantification of the particle shape of three fractions of carbonate sand were conducted for each of the sample particles via software image processing. In terms of particle strength tests, particular emphasis was placed on examining the effect of particle size and coordination number on particle strength, and the number of particles tested is summarized in Table 1. The sand was sieved into three uniform fractions, namely 5–10 mm, 2–5 mm, and 1–2 mm (denoted as A, B, and C, respectively, in the specimen code). Different multiple discrete contact numbers (i.e., the coordination number) of particles directly affect the crushing strength of particles. In order to examine the effect of coordination number (CN) on particle strength, single-particle compression tests were conducted under three coordination numbers, i.e., CN2, CN4, and CN6. In a regular single particle compression test, the particle is compressed by top and bottom platens, during which two contact points were created ideally from the top and bottom directions, i.e., coordination number is nominally two. A higher coordination number was achieved by creating extra contact points with quartz sand particles. Relatively spherical and rounded quartz sand particles that are similar in size with the specimen tested were selected and glued to loading platens with high stiffness epoxy resin, which ensures that the particles will not slide and change the coordination number of particles during compression. The final three-dimensional illustration of CN4 and CN6 test configurations is illustrated in Figure 3a and Figure 4. When the particle is crushed between three fixed quartz sand particles at the bottom and one at the top, the test is a multi-particle crushing test with a CN equal to 4; moreover, when there are three particles at both the bottom and the top, the test used is the multi-particle crushing test with a CN equal to 6. In order to ensure that the strength of the bottom and top particles is strong enough so as to not break in the compression process in advance, these particles adopt quartz sand with higher strength and are replaced after each compression test. The carbonate sand particle was supported by quartz sand particles at the lower platen and compressed against quartz sand particles on the top platen during the test. It is worth noting that given the irregular and elongated particle shape of the carbonate sand, the bottom panel, which was set to be laterally unconstraint, was adjusted before the test to ensure a designated number of contacts and a firm constraint condition to avoid undesirable sliding. In total, 390 particles were subjected to particle compression tests, and the particles are divided into nine categories with detailed testing program provided in Table 1. Around 45 particles were tested for each category.

### 2.4. Particle Shape Parameters Characterization

Digital microscopes were utilized to observe particles and capture images for further particle shape and morphology analyses. For each of the particles, two images were taken from the front and top view, and particle shape parameters were analyzed from the two perspectives. The scale of images taken from the digital microscopes was calibrated with a reference gauge; thus, the dimensions of the particles can be accurately derived from images. The images captured were first binarized before seven fundamental dimensions were yielded from image analysis, which includes, perimeter (*P*), area (*A*), maximum Ferret diameter (*D_Fmax_*), minimum Ferret diameter (*D_Fmin_*), circle diameter (*D_cm_*), the perimeter of the external polygon (*P_c_*), and perimeter of the ellipse (*P_e_*). The dimensions of the fundamental size parameters measured for a representative particle are marked and illustrated in Figure 4, and the corresponding detailed definitions of the fundamental size parameters are listed in Table 2. Based on the fundamental dimensions, a set of particle shape parameters can be derived, namely area aspect ratio (*AR*), area sphericity (*S_A_*), roundness coefficient (*R_c_*), roughness (*R_g_*), and angularity (*A_g_*), and their definitions are subsequently provided.

Aspect ratio (*AR*), which represents the elongation property of the particles, is defined as the ratio of the maximum Ferret diameter to the minimum Ferret diameter:

(1) $$AR=\frac{D_{F_{min}}}{D_{F_{max}}}$$

where *D_Fmax_* is the maximum Ferret diameter, and *D_Fmin_* is the minimum Ferret diameter. It is a commonly used particle morphology descriptor and ranges between 0 and 1 [32]. The flatter and narrower the particles, the small the value; conversely, the closer the particle contour to the circle, the closer the value to unity.

Area sphericity (*S_A_*) describes the similarity of a particle shape to a sphere, which is defined as the ratio of the area of the particle (*A*) to the area of the smallest circumscribing circle ($\pi D_{cm}^{2}/4$) [33]. The more irregular the particle shape, the smaller the sphericity value and the closer it is to zero. The more spherical the particle shape is, the closer the sphericity value is to one:

(2) $$S_{A}=\frac{A}{\pi D_{cm}^{2}/4}$$

where *A* is the area covered by the particle profile, and *D_cm_* is the minimum inner diameter of the smallest circumscribed circle.

Roundness coefficient (*R_c_*) describes whether the particle is circular, and *R_c_* equals to 1.0 for a perfectly circular particle. In this study, roundness coefficient (*R_c_*) adopts the simplified calculation method, as proposed by Kato et al. [26] and Xu et al. [34]. The roundness coefficient is defined as follows:

(3) $$R_{c}=\frac{P^{2}}{4\pi A}$$

where *P* is the perimeter of the particle profile, and *A* is the area covered by the particle profile.

Roughness (*R_g_*) reflects the roughness of the particle’s surface. The direct measurement of roughness is difficult because its value is scale dependent [35]. At the micro-scale observation down to the nanometer level, surface roughness can be measured, whereas roughness in this study refers to the square of the ratio of the perimeter of the particle to the minimum perimeter of the circumscribed polygon at the meso-scale [36]:

(4) $$R_{g}={\left(\frac{P}{P_{c}}\right)}^{2}$$

where *P_c_* is the perimeter of the convex outline of the object. The more complex the grain boundary curve, the greater the roughness becomes and vice versa.

Angularity (*A_g_*), characterizes the number and protrusion degree of edges and corners of the particles [36]. The larger the number of edges and corners and the greater the distance of the protruding particle edge, the greater the angularity. The angularity is defined as follows:

(5) $$A_{g}=\frac{P_{c}}{P_{e}}$$

where *P_c_* is the perimeter of the convex outline of the object, and *P_e_* is the perimeter of the equivalent ellipse.

## 3. Results and Discussion

### 3.1. Analysis and Discussion of the Particle Shape Parameters

**Figure 5:**
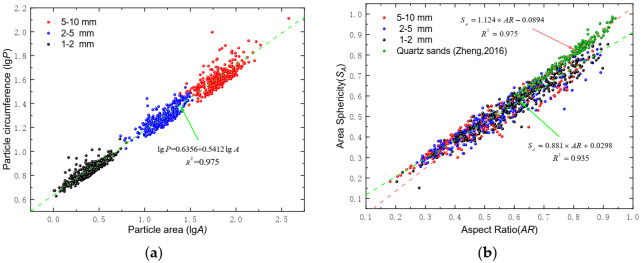
(**a**) lgP–lgA fitting curve of carbonate sand; (**b**) AR-SA fitting curve of carbonate sand.

When analyzing the relationship between the area and perimeter of the particles at log-scale, a linear correlation can be drawn, which follows the fractal concept. From a two-dimensional perspective of morphology, the larger the perimeter of the particle, the larger the area. In the same perimeter, the area decreases with an increase in regularity of the particle shape. It can quantify the effect of irregular particle shapes by the fractal dimension (*D_R_*) of particles [37]. *D_R_* is defined as half the slope of the best-fit line of the lg *P*-lg *A* in Equation (6) [38]:

(6) $$\mathrm{lg}P=k+\frac{D_{R}}{2}\mathrm{lg}A$$

where *k* is a constant. The more complex the particle profile, the larger the fractal dimension is, and the more irregular the particle shape will be. Figure 5a shows the fitting linear relationship between the natural logarithm of area and perimeter on 390 carbonate sands. The area and perimeter of particles increased as particle size increased; however, the fractal dimension among the three fractions appeared to be constant. The fractal dimension of the carbonate sand is quantified to be 1.08, which is close to what was reported by Wang et al. [36] on carbonate sand, while for quartz sands *D_R_* was reported to be between 1.0 and 1.035 [39].

A linear correlation could be found between the area sphericity and aspect ratio. Figure 5b illustrates that a linear correlation could be drawn between the area sphericity and aspect ratio of the three fractions of the carbonate sand with limited scatter in data. The aspect ratio and area sphericity of different fractions fall in similar regimes, i.e., the *AR* values varied between 0.181 and 0.892, with a mean value of 0.533, and *S_A_* values are within the range of 0.151 to 0.861. The best fitted linear relationship is provided by the following equation.

(7) $$S_{A}=0.881\times AR+0.0298$$

Studying Jordan Formation frac sand and quartz sand from the USA, Zheng and Tannant [40] reported a similar linear relationship between sphericity and aspect ratios with a higher slope value, and the results are plotted in Figure 5b for comparison purposes. The aspect ratio and area sphericity of the carbonate sand is distributed in a much lower and wider range, compared with those of frac sand, which is relatively rounded in shape. Compared with quartz sands, some researchers mentioned that carbonate sand particles are, in general, more irregular and angular in shape [1,6,9,41], and the aspect ratio of them are markedly lower from the analysis in Figure 5b, which indicates the high frequency of platy or elongated carbonate particles. The ratio between *S_A_* and *AR* can be rewritten as follows based on their definition provided in Section 2.

(8) $$\frac{S_{A}}{AR}=\frac{4A}{\pi D_{cm}^{2}}\times \frac{D_{F_{max}}}{D_{F_{min}}}$$

From Equation (8), it was indicated that for ideal elliptical particles, the ratio should equal to unity, whereas, in reality, the maximum ferret diameter *D_F_*_max_ and the major axis *D_cm_* are not equal. Thus, the irregularity of carbonate sand particles is the major reason for the deviation of the slope from unity.

**Figure 6:**
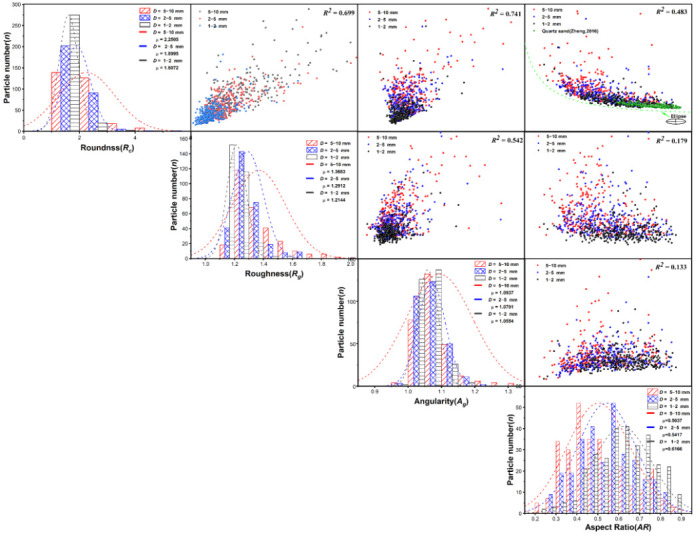
The distribution of four particle shape parameters and the relationships between each two of the parameters.

The distributions of four particle shape parameters, i.e., roundness, roughness, angularity, and aspect ratio, are plotted in Figure 6 in terms of the statistical histogram and best-fitted normal distribution probability density curves, and the relationships between the two parameters are presented in the subfigures on the top right. The distributions of the particle shape parameters are scattered, and there is no strong correlation between the parameters. As shown by the bar charts in Figure 6, the angularity, roughness, and roundness of calcareous sand are roughly positively correlated with each other, while slight negative correlation can be concluded between each of the three parameters and the aspect ratio. The distributions of the smaller-sized fractions are more concentrated, with relatively narrower probability density curves and lower standard deviations based on normal distribution fitting. The angularity, roughness, and roundness of calcareous sand have an increasing trend with an increase in particle sizes, while the trend of the aspect ratio is completely opposite and an average aspect ratio of the 5–10 mm fraction is the highest among the three fractions.

**Figure 7:**
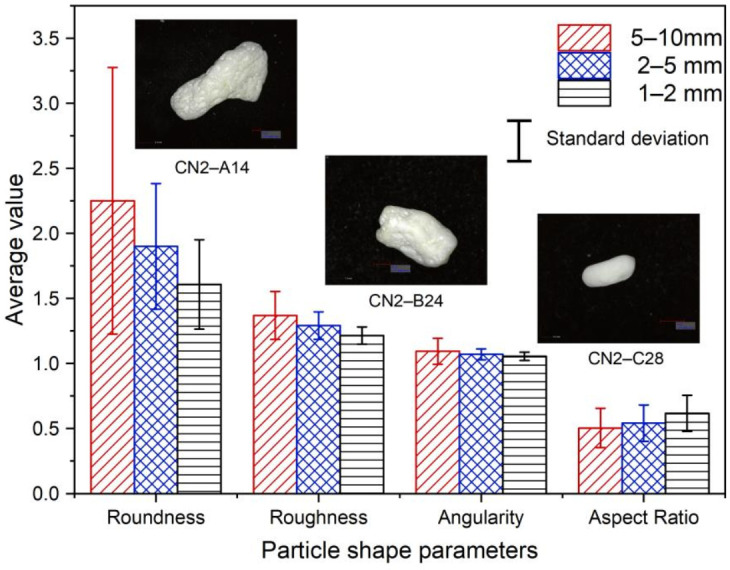
Average particle shape parameters against the mean grain size plot.

Figure 7 provides average and standard deviation values for roundness, roughness angularity, and aspect ratio, from which it is indicated that calcareous sand with smaller particle size has lower roundness, roughness, and angularity values, i.e., the particles are closer to circular with a smoother surface. Three representative particles from the different size fractions are also provided in Figure 7, where the particles become smoother and regular with less sharp edges and defects as the particle size decreased from 5–10 mm to 1–2 mm. From the perspective of shape, the aspect ratio of massive, flake, and spindle particles are generally between 0.4 and 1, while the aspect ratio of dendritic and rod particles is less than 0.4. It can be observed that the aspect ratio of 5–10 cm in calcareous sand is less than 0.4, with more dendritic and rod particles. Given the origin of carbonate sand, large calcareous sand particles retain some structural characteristics of protozoa (i.e., coral, algae, and shell), resulting in highly irregular particle shapes. Small-sized calcareous sand particles are products of crushing experienced by large-size coral sand particles due to weathering and transportation and during which the corners and edges of the particles are often broken and ground. Thus, the particles are less elongated and more regular in shape.

### 3.2. Particle Strength Characteristics and Influencing Factors

**Figure 8:**
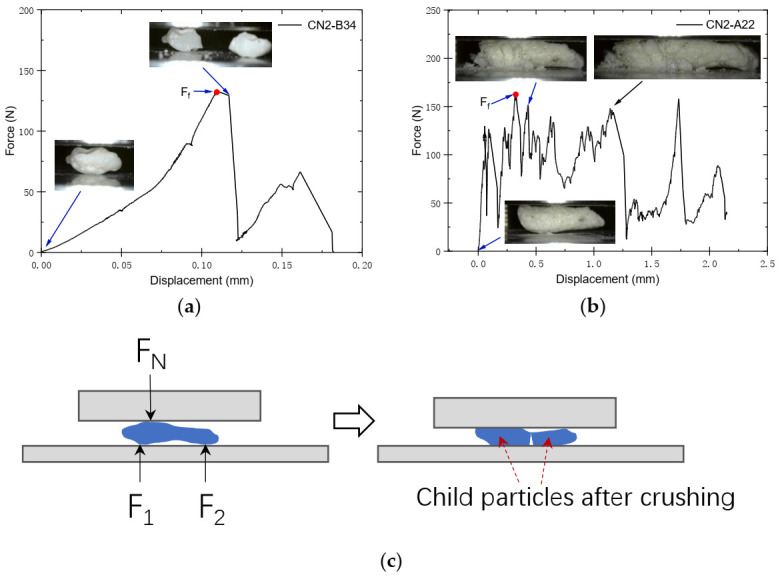
(**a**) Typical splitting mode failure; (**b**) typical progressive fracture mode failure; (**c**) elongated particle breakage schematic diagram.

Two brands of particle failure modes can be concluded from the particle crushing tests, i.e., the splitting mode and the progressive fracture mode. Typical force–displacement curves of the two failure modes of the carbonate sand are plotted in Figure 8a,b, with the images of the particles in different stages of the tests presented. Particles with higher area sphericity values were observed to possess a higher possibility of splitting failure, while elongated particles were more likely to fail progressively. The irregular morphology of sand particles would affect stress distribution in particles during compression, which is believed to be the major cause of the difference in terms of the fracture modes [29,42]. It is worth noting that for elongated and irregular particles, such as carbonate sand, the coordination number equaling to two constraint conditions is not easily achievable. When tested with only the top and bottom platens, where the coordination number should nominally be two, the elongated particles are often observed to have multiple contact points instead of two against the loading platens along the long axis under compression, which results in bending moments being applied to the particle, as presented by the simplified schematic illustration in Figure 8c. Due to the low aspect ratio of carbonate sand particles, especially for large-sized particles, this type of bending failure continuously happens to the fractured particles until the child particles are usually still elongated. Thus, a saw tooth type of force–displacement curve with high fluctuation magnitudes (Figure 8b) was yielded, while splitting mode failure provided a relatively smooth loading curve with a distinct abrupt failure point.

According to the definition of Jaeger [43], the compressive strength of a single particle can be calculated as follows.

(9) $$\sigma =\frac{F_{d}}{d^{2}}$$

$F_{p}$ is the failure force, and d is the particle diameter. It is generally taken as the minimum and intermediate particle dimension in order to be more representative of the tension area.

**Figure 9:**
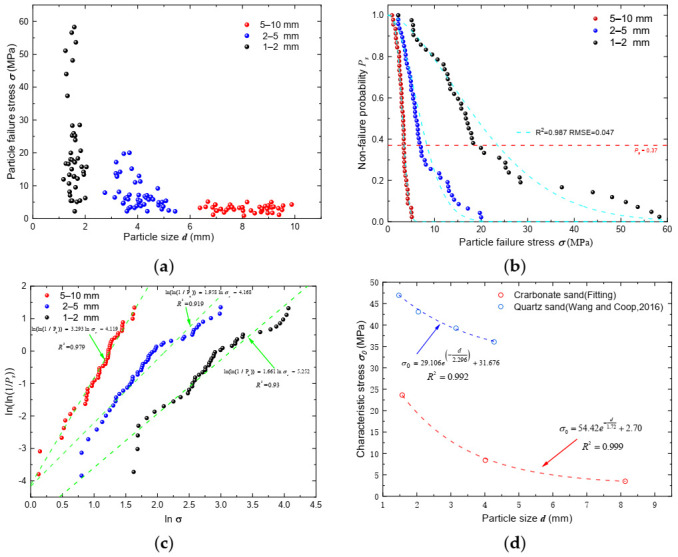
(**a**) Failure stress versus particle size plot; (**b**) non-failure probability distribution curve; (**c**) Weibull distribution fitting results; (**d**) characteristic stress versus particle size plot for the three fractions of carbonate sand.

The particle failure stress is plotted against the particle diameter for each individual specimen tested in Figure 9a, and particle strength distribution curves of the three fractions are presented in Figure 9b in terms of the non-failure probability against particle peak stress. Notable size effects can be concluded from the figures. Failure stress, as well as the variability of data, decreases dramatically with an increase in particle size in general. Statistical analysis linking the probability of failure with failure stress proposed by Weibull [44], followed by McDowell and Bolton [45], was utilized to analyze and describe particle strength distribution characteristics quantitatively. The relationship between the survival probability of a particle and the tensile stress can be described by Weibull statistics:

(10) $$P_{s}=\exp\left[-{\left(\frac{\sigma -\sigma_{u}}{\sigma_{0}}\right)}^{m}\right]$$

where *P_s_* is the particle’s non-failure probability, m is the Weibull modulus, $\sigma_{0}$ is the characteristic stress, which corresponds to the particle failure stress when *P*_s_ is equal to exp^−1^ (≈0.37), and $\sigma_{u}$ is the stress at zero probability of failure. For brittle materials, $\sigma_{u}$ is often taken as zero. Based on experiments, the non-failure probability at the given characteristic stress *σ* can be calculated based on the following:

(11) $$P_{s}=\frac{n}{N}$$

where n is the number of surviving particles at the given stress, and N is the total number of particles. For the ease of determination of the Weibull modulus, Equation (10) can be transformed to Equation (12) by taking the logarithm of both sides twice:

(12) $$\ln\left[\ln\left(\frac{1}{P_{s}}\right)\right]=m\ln\left(\frac{\sigma }{\sigma_{0}}\right)=m\ln\sigma -b$$

where $b=m\ln\sigma_{0}$. The Weibull modulus interprets the variability of the particle strength, and the characteristic stress mainly reflects the strength of particles. As shown in Figure 9c, the *m* and *σ*_0_ values are determined by the best-fitted curves, and the corresponding fitted equations are provided alongside the curves following Equation (12). Note that some researchers, e.g., Wang and Coop [29], adopted experimental characteristic stress during the fitting process. However, *σ*_0_ was considered as a variable constant, apart from the constant *m*, during the fitting process in this study, which yielded much higher *R*^2^ values; meanwhile, the fitted curves in Figure 9b did not converge with the experimental data at *P_s_* = 0.37. The characteristic strength of particles can be obtained, and all parameters are summarized in detail in Table 3.

The Weibull modulus of the three fractions decreased as particle size increased, which indicates that the failure stress of larger fractions was more concentrated. Characteristic stress, on the other hand, decreased markedly as particle size increased (Figure 9d), and the relationship follows the exponential relationship as follows.

(13) $$\sigma_{0}=54.42e^{-\frac{d}{-1.72}}+2.70$$

Particle size effect on particle strength agreed well with some literature work [29,46,47]. Lower characteristic stress and higher Weibull modulus were also found for relatively larger sized quartz particles by Coop [29], and the fitted equation for the quartz sand is also given in Figure 9d [29]. Particle size is the major factor that affects the strength of brittle materials. Pino and Baudet [48] speculated that the increased granule size results in an increase in the probability of failure because more flaws exist in a larger sample, while the small-sized quartz sand particles fail with both splitting and explosive modes, which resulted in a variation in strength results [29]. Similar results can be found when particles with a small grain size produce wider Weibull distributions [49,50]. The decreasing trend of both curves is quantitatively similar, although the magnitude of the characteristic stress for the quartz sand is much higher than that of carbonate sand. Intra-particle voids and fissures are distributed in carbonate sand particles, and the strength of the particles, as well as fracture patterns, are dominated by the internal void that would induce possible coupled bending, shear, and tensile cracks during the particle breakage process [30]. Therefore, carbonate sand is much more crushable, with notably lower particle strength compared with regular quartz sands.

### 3.3. Influence of Coordination Number on Particle Strength

**Figure 10:**
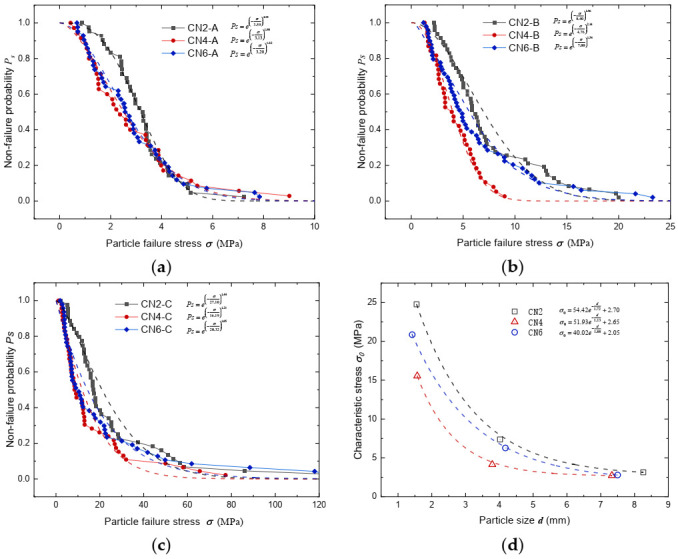
Effect of coordination number on particle non-failure probability distribution curves for (**a**) fraction 5–10 mm, (**b**) fraction 2–5 mm, and (**c**) fraction 1–2 mm; (**d**) characteristic stress against particle size plot for different coordination number testing scenarios.

Grains within a loosely packed matrix have a lower coordination number, whereas grains within a more densely packed matrix have a higher coordination number [51,52]. The influence of discrete contact on isolated crushing is studied by changing the number of support particles to change the number of contacts, which is the coordination number, in order to mimic real assembly conditions at grain scale. The effects of the coordination number on particle strength distribution curves of the three fractions are illustrated in Figure 9b,c and Figure 10a, from which it is observed that the particle strength at the CN2 scenario always excelled those of the CN4 and CN6 scenarios. Interestingly, particle strength appeared to be at its lowest when the coordination number equaled four. The detailed characteristic stress and Weibull modulus fitted are summarized in Table 3. The effect of coordination number on carbonate sand particle strength was very different from what was reported by Todisco et al. [28], who tested Leighton Buzzard sand and crushed limestone particles. Todisco et al. [28] reported a consistent increase in particle strength with an increase in the number of contacts for both granular materials they tested. The markedly different behavior between carbonate sand particles and the sand tested by Todisco et al. [28] is believed to be mainly attributed to the drastic difference in their aspect ratios. The extraordinarily low aspect ratio values found from carbonate sand particles can result in high tensile stress concentration in higher coordination number scenarios. For example, as illustrated in Figure 3, in the CN4 loading scenario, the elongated particle is usually supported by three distributed contact points from the bottom while being loaded by a single loading point from the top. As the loading points are not aligned, extra tensile stress is concentrated on the bottom side of the carbonate sand particle, which decreased the overall particle strength (Figure 10). Therefore, in the case of carbonate sand, the extra contact points did not supply extra support or decrease the tensile stress; thus, particle strength in the CN4 scenario appeared to be the lowest. This experimental finding agreed well with the theoretical analysis conducted by McDowell et al. [45].

The coupled effect of particle size and coordination number on the characteristic stress is illustrated in Figure 10d, from which it is observed that the particle size is still the main factor affecting particle strength. Under the same particle size, the strength of CN2 is the highest, but that of CN4 is the lowest, while that of CN6 is close to and slightly higher than that of CN4. The effect of particle size on characteristic stress for CN4 and CN6 tests also can be presented by exponential equations as follows.

(14) $$\sigma_{0}=51.93e^{-\frac{d}{1.13}}+2.65$$

(15) $$\sigma_{0}=40.02e^{-\frac{d}{1.88}}+2.05$$

### 3.4. Influence of Morphological Parameters on Particle Strength

**Figure 11:**
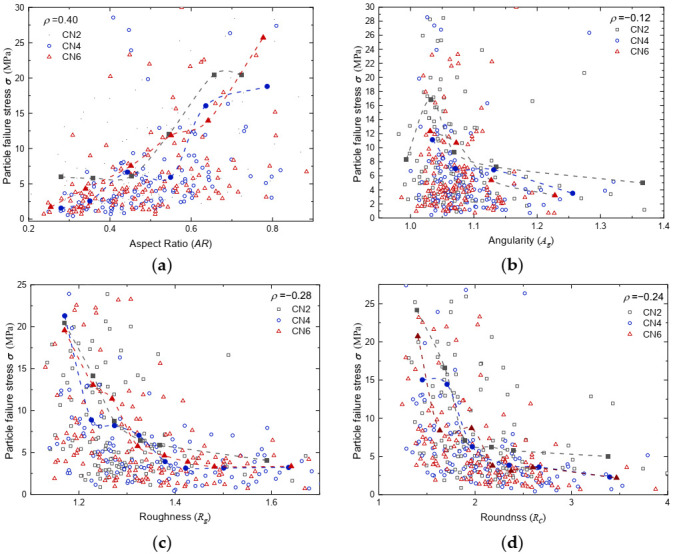
(**a**) The effect of aspect ratio on failure stress; (**b**) the effect of angularity on failure stress; (**c**) the effect of roughness on failure stress; (**d**) the effect of roundness on failure stress.

The relationships between the particle strength and representative particle shape parameters, i.e., aspect ratio, angularity, roughness, and roundness, are provided in Figure 11. Both original data points (illustrated by hollow markers) and average values (illustrated by solid markers connected with dashed curves) are plotted in the figures. Despite scatter, failure stress notably increased with an increase in aspect ratio and/or a decrease in angularity, roughness, and roundness based on the average lines. This indicates that regularly shaped particles are more likely to yield higher particle strength and integrity, while elongated and irregular particles are more likely to be easily crushed. This matched with the discussion in Section 3.2. The regularly shaped particles are more likely fail following the split mode, while elongated particles generally progressively fracture under compression. Wang and Coop [29] reported that there is a strong correlation between particle crushing strength and local morphological parameters, and this was found to be true for carbonate sand as well from the results presented in Figure 11. The scatter of data was expected, which originated from the interparticle variation nature of geomaterials, e.g., the distribution of intra-particle voids or mineralogy, possible fissures or cracks, and structural flaws. A weak correlation between particle peak strength and morphological parameters for carbonate sands was also reported by [31].

## 4. Conclusions

The particle strength of three uniform fractions (5–10 mm, 2–5 mm, and 1–2 mm) of carbonate sand from the South China Sea was tested under three constraint conditions (i.e., coordination number equals to 2, 4, and 6). A series of particle shape descriptors was analyzed, and their impact on particle strength was investigated. The major conclusions of the current study are as follows:

1. The particle shape parameters of particles show a trend of normal distribution. With an increase in particle size, roundness (increased from 1.61 to 2.25), roughness (increased from 1.21 to 1.37), and angularity (increased from 1.05 to 1.09) gradually increase, while aspect ratio decreases from 0.62 to 0.50. This shows that the irregularity and angularity of particles decreased with a decrease in particle size. Compared with quartz sand (i.e., 1.0–1.035), in the literature, the fractal dimension of calcareous (i.e., 1.0–1.035) sand is larger, which proves that its shape is more irregular than that of quartz sand.
2. Carbonate sand presents two-particle crushing modes, i.e., splitting mode and progressive fracture mode, which will affect stress distributions. The mean particle strength of carbonate sand of different sizes is 3.49 MPa–23.62 MPa, which is much lower than that of ordinary quartz sand (i.e., 36.5 MPa–46.93 MPa). This can be because more voids and defects can be found in larger-sized calcareous sand particles. Similar to the results of quartz sand, although the relationship between particle strength and particle size has high dispersion, it showed an exponential relationship for average values. The smaller the particle size, the higher the particle strength.
3. The coordination number, in general, did not affect markedly the particle strength, but particle strength exhibited the highest and lowest crushing strength at CN2 (i.e., 3.49 MPa–23.62 MPa) and CN4 (i.e., 2.73 MPa–15.53 MPa). This is drastically different from what was reported for quartz sand and crushed limestone particles. Although data showed a certain degree of scattering, the strength of the particles decreased with an increase in roundness, roughness, and angularity and a decrease in aspect ratio, which manifests that the irregularity of particles will reduce the particle’s strength.

## Figures and Tables

**Table 1:** Summary of carbonate sand particles tested.

Specimen Code	Particle Size	Coordination Number	Number of Particles	
CN2-A	5–10 mm	2	43	120
CN4-A	5–10 mm	4	35	
CN6-A	5–10 mm	6	42	
CN2-B	2–5 mm	2	47	134
CN4-B	2–5 mm	4	38	
CN6-B	2–5 mm	6	49	
CN2-C	1–2 mm	2	44	136
CN4-C	1–2 mm	4	46	
CN6-C	1–2 mm	6	46	

**Table 2:** Definition of fundamental dimensions of particles.

No.	Symbol	Basic Size Parameters	Desciption
1	*P*	Perimeter	The perimeter of the particle profile
2	*A*	Area	The area covered by the particle profile
3	*D_Fmax_*	Maximum Ferret diameter	Maximum distance between two boundary parallel lines of a particle projection contour
4	*D_Fmin_*	Minimum Ferret diameter	Minimum distance between two boundary parallel lines of a particle projection contour
5	*D_cm_*	Circle diameter	Minimum inner diameter of the smallest circumscribed circle
6	*P_c_*	Perimeter of external polygon	Perimeter of the convex outline of the object
7	*P_e_*	Perimeter of ellipse	Perimeter of the equivalent ellipse

**Table 3:** Weibull distribution fitting parameters.

Specimen Code	*d*	$\bar{\sigma }$	*m*	*b*	$\sigma_{0}$	*R* ^2^
CN2-A	8.26	3.14	3.29	4.12	3.49	0.98
CN2-B	4.02	7.37	1.96	4.17	8.40	0.92
CN2-C	1.55	24.76	1.66	5.25	23.62	0.90
CN4-A	7.33	2.73	1.67	1.90	3.13	0.96
CN4-B	3.79	4.14	2.14	3.34	4.76	0.95
CN4-C	1.57	15.54	1.21	3.36	16.19	0.88
CN6-A	7.5	2.79	1.82	2.12	3.20	0.96
CN6-B	4.18	6.27	1.54	3.00	7.00	0.99
CN6-C	1.42	20.85	1.05	3.17	20.32	0.83
